# Perioperative Analgesic Effect of Serratus Anterior Plane Block for Breast Surgery: A Randomized Control Study at a Large Teaching Hospital in Ghana

**DOI:** 10.7759/cureus.63397

**Published:** 2024-06-28

**Authors:** David K Mensah, Papa Kobina G deGraft-Johnson, Ebenezer O Darkwa, Alexander Akowuah, Owusu-Sekyere Danso, George Aryee, Raymond Essuman, Robert Djagbletey

**Affiliations:** 1 Anaesthesia, Intensive Care and Pain Management, Korle-Bu Teaching Hospital, Accra, GHA; 2 Department of Anaesthesia, University of Ghana Medical School, Accra, GHA

**Keywords:** serratus anterior plane block, ponv, pain management, regional anaesthesia, mastectomy

## Abstract

Background

Pain after breast surgery has been described as moderate to severe in intensity and, if inadequately treated, increases postoperative morbidity, hospital cost, and the incidence of persistent postoperative pain. Serratus anterior plane (SAP) block is an interfascial injection technique for analgesia of the chest wall. There is a lack of data with regard to its analgesic and possible opioid-sparing effects in Sub-Saharan Africa. This study aimed to determine the perioperative analgesic effect of serratus anterior plane block administered for breast surgery.

Methods

This was a prospective, randomized, double-blinded study involving 52 patients and was randomized into the intervention (n = 26) and control (n = 26) groups. One patient in the control group did not receive the allocated intervention, while one in the intervention group lost to follow-up. Complete data of 50 participants, comprising intervention (n=25), was used in the analysis.

Patients’ demographic and health characteristics, pre-induction, intra-operative, and postoperative hemodynamic parameters were noted. After induction of anesthesia, a blinded anesthetist performed an ultrasound-guided serratus anterior plane block with 0.25% plain bupivacaine or a sham block using 0.9% normal saline (control). Numerical rating scale (NRS) score and incidence of postoperative nausea and vomiting (PONV) were recorded immediately after surgery and at 1, 4, 8, and 24 postoperative hours. Patient satisfaction with analgesic management within the first 24 postoperative hours was also assessed.

Results

Patients who received SAP block had lower NRS scores at all measured time points, but this was only statistically significant at the fourth postoperative hour (*p-value *= 0.002).

Compared to controls, patients who received SAP had lower intraoperative (11.3±1.5 mg vs. 11.9±1.5 mg, *p value *= 0.131) and postoperative (4.6±5.7mg vs. 10.5±6 mg, *p value*=0.001) mean opioid consumption. However, only the reduction in postoperative opioid consumption was found to be statistically significant.

Most participants (> 90%) in this study did not experience PONV and were very satisfied with their postoperative pain management.

Conclusion

Serratus anterior plane block reduces NRS pain scores postoperatively. It also significantly reduces postoperative opioid consumption but does not significantly reduce intraoperative opioid consumption.

## Introduction

Breast cancer is the most common cancer in women and is a major public health problem worldwide [1]. Management is multidisciplinary and usually involves a combination of surgery, radiotherapy, and chemotherapy [2]. For the majority of breast cancer cases, surgery is the primary and most effective therapeutic intervention [3]. Breast surgery is an integral part of the management of breast cancer, and it covers a wide range of procedures such as lumpectomies, mastectomy with axillary clearance, and reconstruction.

The majority of patients who undergo breast surgery have been found to experience severe acute postoperative pain [4]. Mastectomy, the mainstay of treatment for patients with breast cancer at the study site [5], is frequently associated with significant postoperative pain, nausea, and vomiting, which lead not only to increased patient suffering but also to a prolongation of hospital stay and increased health-related costs [6]. Pain management at the study site, which is mainly opioid-based, is reported to be unsatisfactory and associated with deleterious effects, including an increased incidence of postoperative nausea and vomiting (PONV) [7].

A modality that provides good postoperative analgesia after breast surgery with minimal side effects will enhance patient care at the Korle Bu Teaching Hospital. 

The serratus anterior plane block has recently been described and reported to provide analgesia to the anterolateral chest wall by blocking the lateral branches of the intercostal nerves [8]. It has rapidly gained popularity due to its relative simplicity, safety, and perceived efficacy [9].

There is a lack of data with regard to the analgesic and possible opioid-sparing effect of serratus anterior plane block in the Sub-Saharan African population. This study therefore sought to determine the perioperative analgesic effect of serratus anterior plane block in patients undergoing elective breast surgery at the Korle Bu Teaching Hospital, Accra, Ghana.

## Materials and methods

Study design

This was a prospective randomized double-blind placebo-controlled study.

Study site

The study was conducted over 12 months at the surgical block of the Korle Bu Teaching Hospital, a major tertiary and largest referral center in Ghana. The hospital has approximately 2000 bed capacity and 23 theaters. Breast surgeries are mainly done by the breast surgery team which has two consultant surgeons and two senior residents who performed the surgeries in this study. The surgeries were performed on the main first-floor surgical theater suite.

Study population

Patients aged 18 to 70 years with an American Society of Anaesthesiologists (ASA) physical status I and II scheduled for elective breast surgery who gave written informed consent were consecutively recruited. Patients with known allergies to bupivacaine, bleeding disorders, infection in the posterior and mid-axillary region, weight less than 50 kg, obesity (BMI >35 kg/m2), and pregnant patients were excluded.

Sample size

In a similar study, Rahimzadeh et al. found that six hours postoperatively, the mean VAS (µ1) was 2.3 with a standard deviation (σ1) of 0.3 in the intervention group and 2.6 (µ2) with a standard deviation (σ2) of 0.6 in the control group [10]. Based on a power of 90% and a confidence level of 95%, the sample size of 50 was found to be adequate using the formula by Sharma et al. [11].

Randomization into groups

Patients were randomized into two groups (Intervention and Control groups) using balloting without replacement the day before surgery during the preoperative visit by the anesthetist.

Blinding

The patients and the investigators were blinded to the study treatments. The study treatments were coded by a consultant anesthetist not involved in the study.

The consultant anesthetist prepared a syringe containing either 0.25% plain bupivacaine or 0.9% normal saline, depending on the group the patient has been randomized to. Both solutions are clear and colorless. The code was only revealed to the investigators after data had been collected and analyzed.

Description of procedure

Patients recruited into the study were assessed preoperatively. During the preoperative assessment, the pain assessment tool (Numerical Rating Scale [NRS]) was thoroughly explained to the study participants.

All the patients were given standardized general anesthesia before the block was performed.

After establishing the Association of Anaesthetists of Great Britain and Ireland (AAGBI) standard monitoring with non-invasive blood pressure (NIBP) cuff, three lead electrocardiograms (ECG), and a pulse oximeter, baseline parameters, heart rate (HR), NIBP, and oxygen saturation (SpO2) were noted. After preoxygenation for 3 minutes, anesthesia was induced with 1-2mcg/kg of IV fentanyl and 2-3mg/kg of IV propofol. The airway was secured with a laryngeal mask airway, and anesthesia was maintained with an isoflurane/oxygen/air mixture. One gram of intravenous paracetamol was administered to all patients after induction of anesthesia. Intra-operative HR, systolic blood pressure, diastolic blood pressure, mean arterial pressure, pulse oximetry, and ECG were monitored every 5 minutes.

With the patient under anesthesia and in the supine position, the upper limb on the side to be blocked was abducted to 90 degrees. Skin preparation was done with a 2% chlorhexidine solution with 70% isopropyl alcohol. Under aseptic conditions, an ultrasound-guided (Butterfly IQ, Butterfly Network, Guilford, Connecticut, USA) serratus anterior plane block was performed in the mid-axillary line at the fifth rib using a 100mm 22-gauge sonovisible block needle (B. Braun, Germany).

The needle was introduced in the fascial plane between the posterior border of the serratus anterior muscle and the surface of the fifth rib using an in-plane technique. Confirmation of the correct needle position was done by injecting 1-2 ml of 0.9% normal saline. After negative aspiration of blood, 0.4 ml/kg of the injectate was administered. Frequent intermittent aspiration was done after delivering 3-5 mls of injectate to avoid intravascular injection.

The deep serratus anterior plane block technique was adopted because it is an easier technique as the rib serves as a reference point for injection and also because it potentially facilitates the blockade of higher-order lateral cutaneous intercostal nerves.

The patient’s vitals were noted immediately after administering the block, at the skin incision, 30 minutes, and one hour after the surgical skin incision. Heart rate and mean arterial blood pressure (MAP) were maintained within 20% of the preoperative baseline values by giving intravenous (IV) bolus doses of 1-2 mg of morphine. Ephedrine 5 mg boluses were given IV as needed to keep MAP greater than 65 mm Hg. Also, atropine 0.5 mg was given IV for a heart rate of less than 50 beats per minute.

At the end of the surgery, patients were extubated fully awake and sent to the recovery ward/post-anesthesia care unit (PACU). Postoperative pain and PONV were assessed as soon as patients were fully conscious and communicating and at 1, 4, 8, and 24 hours after the operation.

One gram of intravenous paracetamol was prescribed six hourly for all patients. Patients with NRS greater than five who were still in the recovery/PACU received intravenous morphine 1-2 mg bolus repeated at 5-minute intervals until the NRS was three or less. Intramuscular pethidine 1-2 mg/kg was given to patients who had been sent to the ward and scored NRS more than five.

The total amount of Morphine and Pethidine administered in 24 hours was recorded. Patient satisfaction with pain management was also assessed 24 hours postoperatively.

Ethical considerations

Approval

The study was approved by the institutional review board (IRB) of the Korle Bu Teaching Hospital (KBTH-IRB/0009/2020).

Informed Consent

Participation in this study was voluntary. All patients who met the inclusion criteria were given detailed information on the purpose, nature, procedures, and potential risks of the study, and informed consent was obtained. Participants were at liberty to withdraw from the study at any time. Data obtained from this study was kept strictly confidential.

Data handling

Data collected was entered into a Microsoft Access database (Redmond, USA). Source document verification was done to ensure accurate and credible data. Data of participants who, after being recruited into the study, were lost to follow-up were censored for removal from data analysis. Participants included in the study were assigned unique identifiers.

Data analysis

Data obtained was exported into and analyzed using IBM Corp. Released 2017. IBM SPSS Statistics for Windows, Version 25.0. Armonk, NY: IBM Corp. Continuous variables were summarized using means and standard deviations, while proportions were used to summarize categorical variables. An independent t-test was used to compare the means of continuous variables between the two groups. A repeated measure analysis of variance (ANOVA) was used to compare the changes in means of continuous variables within the groups. Pearson’s chi-square test or Fisher’s exact test was used to compare categorical variables. A p-value less than 0.05 was considered statistically significant.

## Results

**Figure 1:**
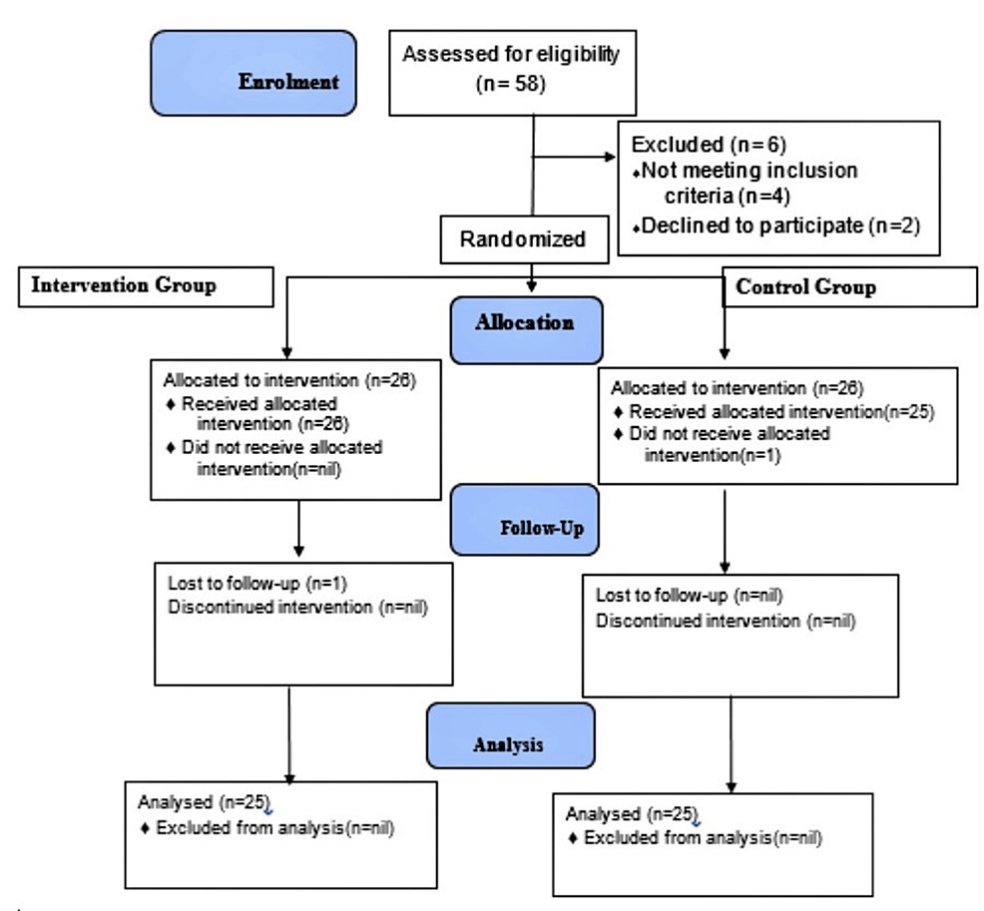
CONSORT diagram

**Figure 2:**
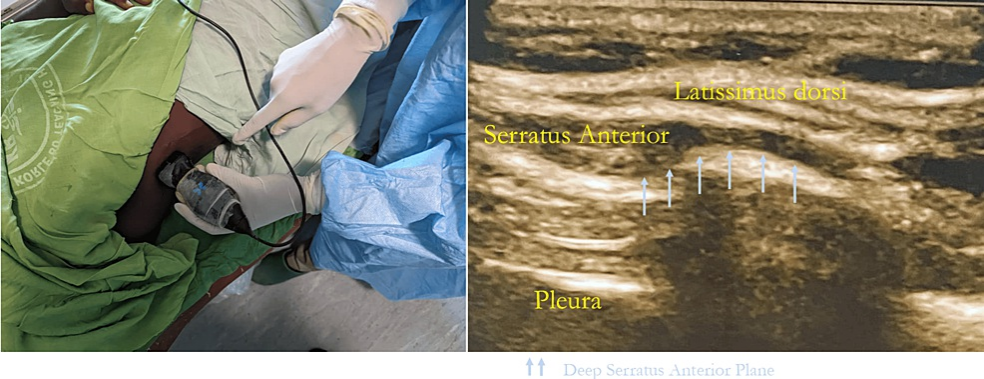
Anatomy of the block along with relevant US images

A total of 52 participants were enrolled in the study and randomized into the intervention (n = 26) and control (n = 26) groups. One patient in the control group did not receive the allocated intervention, while one patient in the intervention group was lost to follow-up. Complete data was obtained for 50 participants, and this was used in the analysis. A CONSORT diagram is provided in Figure 1. The anatomy of the block and US image is shown in Figure 2. 

**Figure 3:**
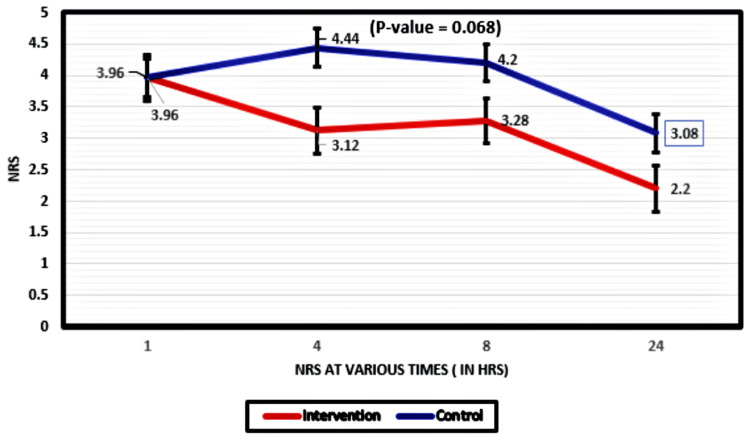
Changes in NRS scores NRS: numerical rating scale, statistical test: repeated measure ANOVA for two factors

The NRS for the intervention group declined from the first hour to the 24th hour, and that for the control group initially increased from the first hour to the fourth hour and then gradually declined. However, the decline in NRS at various times between the two groups was not significant (p-value =0.068) (Figure 3).

Though the mean NRS for the interventional group over the first 24 postoperative hours was significantly lower compared to the control group (p-value = 0.004), the variation in the measured NRS over time between the two study groups was not found to be significant.

The mean total opioids consumed (morphine equivalent) intraoperatively were significantly higher (11.6±1.5mg) compared to opioids consumed postoperatively (7.7±6.5mg). Although not statistically significant, the mean intraoperative opioid consumed (morphine equivalent) was slightly higher in the control group compared to the intervention group.

Patients in the control group consumed a significantly higher mean total opioids (morphine equivalent) postoperatively compared to the interventional group (Table 1).

**Table 1:** Opioids consumed Statistical test: Independent t-test between groups.

	Group (mean±SD)		p-value
	Intervention	Control	
Intraoperative opioid consumed (mg)	11.3±1.5	11.9±1.5	0.131
Postoperative opioids consumed (mg)	4.6±5.7	10.5 ± 6	0.001*

Although the majority of the patients did not experience PONV, there was a 6% and 10% incidence of PONV at four and eight postoperative hours, respectively. There was however no difference in the incidence of PONV between the two groups (Table 2).

**Table 2:** Incidence of PONV and patient satisfaction PONV: Postoperative nausea and vomiting, Statistical test: Chi-squared test performed for PONV between groups. Fisher's exact performed for patient satisfaction between groups.

	Groups: n(%)		p-value
	Intervention	Control	
Incidence of PONV			
1hr			1.000
None	24(96.0)	23(92.0)	
PONV	1(4.0)	2(8.0)	
4hrs			0.098
None	24(96.0)	23(92.0)	
PONV	1(4.0)	2(8.0)	
8hrs			0.349
none	24(96.0)	21(84.0)	
PONV	1(4.0)	4(16.0)	
Satisfaction level			
Very satisfied	19(76.0)	19(76.0)	1.000
Somewhat satisfied	6(24.0)	5(20.0)	
Neutral	0(0.0)	1(4.0)	

There was no significant difference in the level of satisfaction between patients in both groups. The majority of patients were very satisfied with their postoperative pain management. A slightly higher number of patients in the intervention group were somewhat satisfied than in the control group. However, there was no significant difference in the level of satisfaction between the two groups (Table 2).

## Discussion

The study groups were comparable in terms of age, weight, height, and Body Mass Index. Baseline hemodynamic variables were comparable in the two study groups.

The mean age of patients in this study was 47.02±13.67 years. This finding is similar to the study by Brakohiapa et al. [12] in Ghana, where the mean age of patients with breast cancer was found to be 48.7±10 years. Previous reports predict 40 years as the peak age of breast cancer in Sub-Saharan Africa [13-16].

In this study, the mean BMI of the patients was 28 kg/m2, which falls within the overweight category [17]. There have been multiple studies showing a strong association between high body mass index and a higher risk of breast cancer [18-21].

The main indication for breast surgery in this study was breast cancer, which is consistent with the literature [5]. In Sub-Saharan Africa, patients often present late with advanced disease when utilization of other modalities of management may be limited. The Korle Bu Teaching Hospital, being the largest referral center in Ghana, serves the entire country and some patients in the West African sub-region and may account for the high incidence of breast cancer surgery.

This study revealed that modified radical mastectomy is the most performed breast surgery at the study site, with an incidence rate of approximately 60%. This finding is in keeping with findings by Sutter et al. [22] that surgery is the primary form of treatment for breast cancer in Africa. Breast-conserving surgeries and lumpectomies are relatively uncommon [23,24].

The reasons attributed to this are that treatment is often not readily available, delayed, or prohibitively expensive; therefore, patients usually present late with advanced disease. Another factor that was noted by Kantelhardt et al. [25] is that routine screening including mammography and the appropriate resources for appropriate follow-up are not widely available in Africa.

In this study, 64% of patients received neoadjuvant chemotherapy before surgery. There is a huge disparity in chemotherapy rates across the African continent. The percentage of patients receiving neoadjuvant chemotherapy in hospitals in Ghana, South Africa, Nigeria, Cameroon, and Rwanda ranged from 28% to 85%, as reported by Sutter et al. [22].

Some studies have demonstrated serratus anterior plane block to significantly reduce intra-operative opioid consumption [26]. In our study, however, SAPB did not significantly influence intraoperative opioid requirements, a finding similar to that of Nian-Qiang Hu et al. [27].

Our study employed the deposition of local anesthetic deep into the serratus anterior muscle. The spread of local anesthetic in this plane and hence the associated dermatomal block has been reported not to be homogenous [28,29]. Our blocks were performed after the patients were anesthetized; therefore, the adequacy and extent of the block could not be tested preoperatively. Inconsistency in the spread of local anesthetics and the extent of the block may account for our findings.

In this study, SAPB was found to significantly reduce postoperative pain scores, as depicted by a significantly lower mean NRS of the interventional group compared to controls, a finding similar to that made by Bhan et al. [30].

We observed that, though there was a general decline in reported NRS over time in both groups, the NRS of controls increased postoperatively in the first four hours among controls. This might be attributed to the fact that intravenous morphine, the intraoperative analgesic used, has a duration of action of 3-4 hours. Thus, by the fourth hour, its action may have waned, and with the controls lacking an effective regional anesthetic block, this may account for the significant increase in their mean NRS at four hours postoperative.

Abdallah et al. [31] in their randomized control trial did not find SAPB to improve analgesic outcomes in patients who underwent ambulatory breast cancer surgery. However, in this study, we found that SAPB more than halved the total opioids (intravenous morphine equivalent) consumed postoperatively (4.6 mg vs. 10.5 mg, p = 0.001). This is in keeping with the systematic review and meta-analysis by Nian-Qiang Hu et al. [27], which revealed that ultrasound-guided SAPB remarkably decreased the levels of postoperative opioid consumption.

Postoperative nausea and vomiting are some of the most common complications after general anesthesia, causing significant dissatisfaction among patients. According to the literature, 60% to 80% of patients undergoing breast surgery under general anesthesia experience PONV [32]. Traditionally, antiemetics has been the mainstay of management. However, the pharmacological management of PONV is not without adverse effects. Several studies conducted have shown that effective regional anesthesia can reduce the incidence of PONV by reducing the amount of opioids used perioperatively [33,34].

Rodseth et al. [35] in their study have implicated genetic factors in the development of PONV, with Africans experiencing a significantly lower incidence of PONV. Our study used an African population, and this may have contributed to the low incidence of PONV observed.

Most patients in the control group received opioids for pain control around the fourth postoperative hours which might account for the increase in the incidence of PONV observed at this time.

There was no significant difference in the level of satisfaction between the two study groups. Generally, patients in both groups were very satisfied with their postoperative pain management, similar to the findings of Park et al. [26].

Obtaining the NRS score for some of the patients from other West African countries was difficult because of language barriers. Although a good attempt was made in explaining the NRS score to them, it is possible they may have over or underestimated their pain scores.

An accurate assessment of postoperative opioid consumption would have been obtained if patient-controlled analgesia (PCA) equipment were available. This is because some of the ward nurses are sometimes reluctant to give opioids to patients when they request them because of a perceived fear of the patient becoming dependent on them. Unfortunately, PCA devices are unavailable at the study site.

This study was a single-center trial; therefore, caution must be exercised in extrapolating the study findings to the general population. Multiple studies will have to be carried out across the country in order to generalize the research findings.

For those in the intervention group, it was difficult to assess if the block actually worked and also to ascertain the extent of dermatomal coverage. This is because the blocks were performed after patients had had general anesthesia. Perhaps if the blocks were given before induction of anesthesia, then an accurate assessment of all the dermatomes involved would have been known.

A larger sample size may have improved the power of the study and could have resulted in the finding of a significant difference in intraoperative opioid consumption and incidence of postoperative nausea and vomiting. The duration of the Serratus anterior block was not assessed in this study, and this could have impacted the results.

## Conclusions

In patients undergoing breast surgery, serratus anterior plane block results in a reduction of postoperative pain assessed by numerical rating scale (NRS) scores in the first 24-hour postoperative period. SAP block also results in a clinical but not statistically significant reduction in intraoperative opioid consumption. However, it results in a significant reduction in postoperative opioid consumption in the first 24 hours postoperatively compared to placebo. SAP block also decreases clinically the incidence of PONV in patients undergoing breast surgery. However, the reduction is not statistically significant. Patients who had SAP block were generally more satisfied with their postoperative pain management. No patient developed complications related to the application of the block.
